# Epicardial Ablation: Prevention of Phrenic Nerve Damage by Pericardial Injection of Saline and the Use of a Steerable Sheath

**DOI:** 10.1016/s0972-6292(16)30735-5

**Published:** 2014-03-12

**Authors:** Kars Neven, Juan Fernandez-Armenta, David Andreu, Antonio Berruezo

**Affiliations:** 1 Dept. of Rhythmology, Klinik fur Innere Medizin 1, Alfried Krupp Krankenhaus, Essen, Germany; 2 Arrhythmia Section, Cardiology Department, Thorax Institute, Hospital Clínic, Universitat de Barcelona, Barcelona, Spain

**Keywords:** Ablation, epicardium, pericardium, phrenic nerve, saline, steerable sheath

## Abstract

Because of the close proximity of the phrenic nerve to the pericardium, phrenic nerve damage caused by epicardial ablation can easily occur. We report two cases of epicardial VT ablation where pericardial injection of saline, combined with the use of a steerable sheath, successfully prevents the phrenic nerve from being damaged.

## Introduction

In recent years epicardial ablation has become an increasingly used treatment modality for ventricular tachycardia (VT) ablation.[1] Because of the close proximity of the phrenic nerve (PN) to the pericardium, PN damage caused by ablation can easily occur, resulting in PN palsy.[2] The exact course of the PN may be identified by pace-mapping with high-voltage output and use of a 3-dimensional imaging system.[3]

We report two cases of epicardial VT ablation where pericardial injection of saline, combined with the use of a steerable sheath, successfully prevents the PN from being damaged.

## Methods

### Electrophysiologic study and identification of epicardial ablation site

The CARTO electroanatomic mapping system (Biosense Webster, Inc., Diamond Bar, CA, USA) was used to generate epicardial voltage maps of the ventricles. Electrograms with delayed components (E-DCs) in the epicardial scar area were identified. The E-DCs had an activation sequence from the edge to the center of the scar. Therefore, radiofrequency (RF) applications were delivered at the edge of the scar, over E-DCs with the shortest delayed component lateness (i.e., entrance of late potential channels during sinus rhythm), as described previously.[4] Finally, a remap was obtained to establish complete elimination of E-DCs.

### Phrenic nerve mapping

In order to prevent PN injury, after determination of the target ablation site the course of the PN was identified by provoking diaphragmatic stimulation with bipolar pacing at 10 mA and 2 ms pulse width from the distal electrode of the ablation catheter. The locations where the PN could be captured were then marked on the electroanatomic map.

### Injection of saline to separate the phrenic nerve from the epicardium

To increase the distance between the epicardium and the PN, saline was introduced into the pericardium in steps of 20 ml until PN capture was lost. Arterial pressure and fluoroscopy were carefully monitored during and after each injection. After each bolus of saline, pacing was repeated to evaluate diaphragmatic stimulation and therefore PN capture.

### Radiofrequency catheter ablation

RF was delivered using a 3.5 mm Thermo-Cool Navistar catheter (Biosense Webster) and an Agilis steerable epicardial sheath (St. Jude Medical, St. Paul, MN, USA). Energy settings were 40 watt, 45ºC maximum temperature, 17 ml/min ablation catheter flow rate. Successful RF ablation was defined as the complete elimination of all E-DCs and the inability to induce VTs with programmed stimulation. After completion of the RF ablation all fluids were aspirated using a pigtail catheter. Following the procedure, the absence of fluid in the pericardial space was confirmed by transthoracic ultrasound.

## Case report

**Figure 1:**
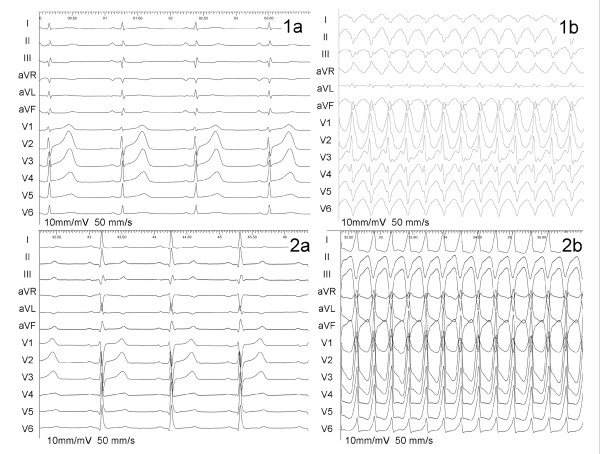
Twelve-lead ECGs. Panel 1a: Sinus rhythm of first patient. Panel 1b: Clinical ventricular tachycardia of first patient. Panel 2a: Sinus rhythm of second patient. Panel 2b: Clinical ventricular tachycardia of second patient.

**Figure 2:**
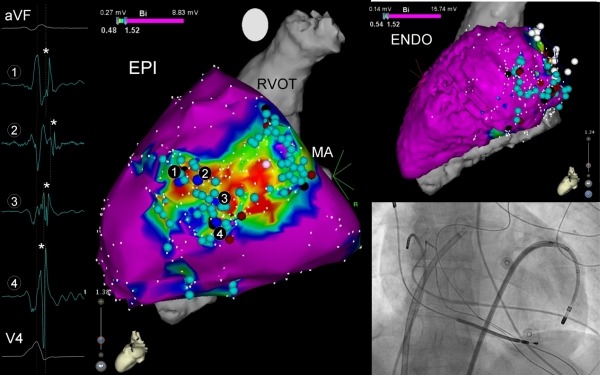
Electroanatomic maps and fluoroscopy of first patient. Electroanatomic map and fluoroscopy of first patient. Left picture shows a left lateral projection of the epicardial, electroanatomic, bipolar LV voltage map. Red, yellow and green colors correspond with areas of low voltage, suggestive of scar tissue. Pink color corresponds with areas of normal voltage, suggestive of healthy cardiac tissue. Light blue dots: E-DCs. Dark blue dots: PN capture. Red dots: ablation points. Black dots near mitral annulus (MA): entry and exit points of the channel. Numbered black dots: EGM of activation pattern through channel, as seen on the left side of the picture (* denotes the delayed component of each electrogram at the corresponding site). Right superior picture shows a left lateral projection of the endocardial electroanatomic bipolar LV voltage map. Light blue dots, E-DCs. White dots, mitral annulus. The endocardial area with the E-DCs corresponds with the epicardial scar area. The endocardium is overall pink, suggesting that there is no scar tissue. Right inferior picture shows a fluoroscopic image in anteroposterior position. From left to right, a dual-coil ICD-lead, a diagnostic catheter in the right atrium, a transseptal steerable sheath, and an epicardial steerable sheath with an ablation catheter during scar mapping can be identified.

**Figure 3:**
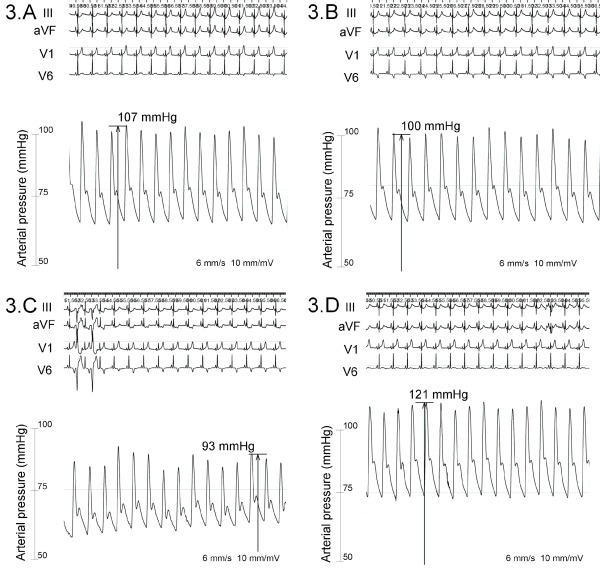
Direct readings of the arterial blood pressure before, during and after saline injection in the pericardium. Panel 3A shows the invasive arterial blood pressure at beginning of saline injection in the pericardium, mean systolic blood pressure is about 107 mmHg. Panel 3B shows the invasive arterial blood pressure during saline injection in the pericardium, mean systolic blood pressure is about 100 mmHg. Panel 3C shows the invasive arterial blood pressure after saline injection in the pericardium and before the start of the radiofrequent ablation, mean systolic blood pressure is about 93 mmHg. Panel 3D shows the invasive arterial blood pressure after complete aspiration of saline in the pericardium, mean systolic blood pressure is about 121 mmHg. All figures show continuous sinus rhythm, figure 3c also shows two ventricular premature beats.

The first patient was a 55-year-old man with no previous cardiac history who suffered three times from syncope. The ECG suggested presence of a VT originating from the LV (Figure 1b). After intravenous administration of amiodarone the tachycardia converted to sinus rhythm with frequent ventricular premature complexes (PVC) of the same morphology as the clinical VT. Echocardiography showed global hypokinesia with LV ejection fraction (LVEF) of 45% and LV end-diastolic diameter (LVEDD) of 57 mm. A coronary angiogram showed no significant coronary artery disease. An electrophysiologic study was performed and the clinical VT was induced. The VT met ECG criteria for an epicardial origin. No ablation was performed. Cardiac magnetic resonance imaging (MRI) showed LVEF of 41% and LVEDD of 58 mm, confirming the results of the echocardiogram. An epicardial scar at the posterolateral basal part of the LV also was identified. Subsequently, a single-chamber implantable cardioverter defibrillator (ICD) was implanted. A second electrophysiologic study was performed as described above and a posterolateral basal scar on the epicardium of the LV was identified, with evidence for E-DCs and two channels going through the scar tissue: one close and parallel to the mitral annulus and the other more apical (Figure 2). The RF ablation of the first channel was successful and uneventful as there was no PN capture. Before starting RF ablation on the site of the second channel, the PN could be captured along the whole trajectory of the identified channel (Figure 2). Without extra precautions RF ablation would have been impossible at these sites. Therefore saline was injected as described above and after injection of 200 ml PN capture was lost. The patient was in sinus rhythm before, during and after the injection, invasive systolic arterial blood pressure remained above 80 mmHg (Figure 3). Then all E-DCs could be eliminated by targeting the late potential channel entrance without any collateral damage. After successful completion of the RF ablation all fluids were aspirated.

**Figure 4:**
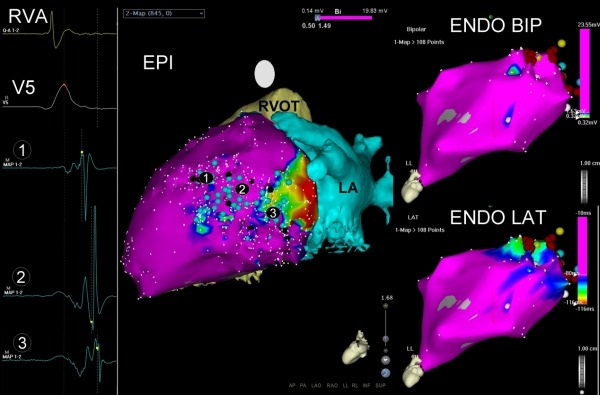
Electroanatomic maps of second patient. Left picture shows a left lateral projection of the epicardial electroanatomical bipolar LV voltage map. Red, yellow and green colors correspond with areas of low voltage, suggestive of scar tissue. Pink color: areas of normal voltage, suggestive of healthy cardiac tissue. Light blue dots: E-DCs. Dark blue dot: PN capture. Black dots: entry and exit points of the channels. Numbered black dots: EGM of activation pattern through channel, as seen on the left side of the picture. Right superior picture shows a left anterior oblique projection of the endocardial electroanatomic bipolar LV voltage map. The endocardium is overall pink, suggesting that there is no scar tissue. Right inferior picture shows a left anterior oblique projection of the endocardial LV local activation time map. No areas have electrograms with delayed components.

The second patient was a 56-year-old man with two previous endocardial VT ablations (posterior fascicular VT two years earlier and focal VT originating from anterior mitral annulus one year before the current events. Recently, ECG had shown polymorphic PVCs and there was an echocardiographic decrease in LVEF from 62% to 40% over 2 years time. A coronary angiogram showed no abnormalities, but a cardiac MRI showed subepicardial basal-lateral LV late enhancement that suggested an area of scarring. An implantable loop recorder showed symptomatic monomorphic sustained VTs. During an electrophysiologic study a VT could be induced (Figure 1d) and an endocardial ablation attempt of the VT in the posterolateral basal part of the LV was unsuccessful. Subsequently, a single-chamber ICD was implanted and an epicardial procedure was scheduled. The identification of the epicardial target ablation site was performed as described above, also showing a posterolateral basal scar on the epicardium of the LV with evidence for E-DCs and one channel going through the scar tissue (Figure 4). Again, before starting RF ablation on the site of the late potential channel entrance, the PN could be captured (Figure 4). Therefore, saline was injected as described above. After injection of 300 ml of saline, PN capture was lost. The patient was in sinus rhythm before, during and after the injection, invasive systolic arterial blood pressure remained above 80 mmHg. All E-DCs could then be eliminated without any collateral damage. After successful completion of the RF ablation all fluids were aspirated.

For both patients the post-procedural course was uneventful and they remained free of PVCs and/or VTs during 6 months of follow-up.

## Discussion

Epicardial ablation is a recent and evolving treatment modality, [1] and serious complications such as damage to the coronary arteries, tamponade and PN damage have been described.[1,2]. In order to prevent complications, some precautions should be taken, such as establishing the proximity of the ablation catheter tip to the coronary arteries, continuous invasive arterial blood pressure monitoring to detect a tamponade, and pace mapping to identify the course of the PN. The consequence of these precautions may be that the electrophysiologist is forced to refrain from ablation because of the increased risk of complications, for example in the case of an ablation site in close proximity to a major coronary artery or when the PN can be captured at the ablation site.[1]

A study by Di Biase et al. compared four methods: inflation of a large balloon in the pericardial space in the area of the PN, introduction of saline, introduction of air and introduction of a combination of air and saline.[5] The techniques used have some disadvantages. Although the method of injecting air in the pericardial space to separate the PN from the epicardium may be practical in itself, there is a potentially dangerous aspect of the method: a layer of air will surround the heart and the defibrillation threshold will be increased because air conducts current only very poorly. Use of a large balloon to separate the PN from the epicardium requires a second pericardial access and positioning can be difficult; the balloon may hinder the placement of the ablation catheter at the desired ablation site.

Di Biase et al. concluded that the combination of air and saline (which causes a so-called "hydropneumopericardium") seemed to be the most effective strategy for preventing PN capture. The combination of air and fluid appeared to allow injection of a higher volume in the pericardial space with a lower impact on blood pressure than use of fluid alone. However, we would like to comment that, according to Boyle's gas law, pressure and volume have a fixed relationship: pV=constant (assuming a constant temperature). Therefore, whether using gas or fluid the volume will be equal at a certain pressure. A much greater volume of air can be introduced in the pericardium is because the air is being compressed by the syringe when introduced in the pericardium. Also, according to Pascal´s law there is no difference in pressure at any point; the pressure in the pericardium is therefore equal at all points. Secondly, a mixture of air and fluid will cause a fluid level. This means that the air and fluid are not mixed and air will accumulate and surround the anterosuperior part of the heart. With the patient being in a supine position during the procedure the air will accumulate between the heart and the defibrillator patches placed on the chest, causing an increased defibrillation threshold in case of defibrillation.

In the study of Di Biase et al. the strategy to prevent PN injury by injecting only fluid failed in all cases although the mean amount of saline infused was 320±30 ml. In our cases we lost PN capture after injection of 200 ml and 300 ml of saline, respectively. The difference between our approach and the approach described by Di Biase et al. is that we used a steerable Agilis epicardial sheath. It is possible that with this steerable sheath the tip of the ablation catheter could be better positioned onto the epicardial surface and thus prevent the capture of the PN.

### Limitations of pericardial injection of saline

1. An iatrogenic tamponade with subsequent hemodynamic instability can occur when injecting fluid into the pericardial space. Adequate safety precautions, such as constant invasive hemodynamic monitoring, should be available before pericardial injection of fluids.

2. Should a ventricular tachycardia occur during catheter manouvering or during ablation sudden hemodynamic intolerance due to the iatrogenic tamponade could occur. Apart from the already mentioned adequate safety precautions, such as constant invasive hemodynamic monitoring, immediate aspiration of the pericardial fluid should be made possible. When before start of the pericardial injection of fluids the epicardial sheath is being connected to a vacuum suction unit by a three-way valve, easy and total aspiration of all epicardial fluids can occur is less than 20 seconds.

3. When injecting fluid into the pericardial space the heart will be compressed. This means that a shift in the electroanatomic map may occur because of the changed position of the epicardium. This does not necessarily have to be a problem, but the electrophysiologist should be aware that the identified target ablation site could have shifted. Remapping of the area of interest is therefore recommended, looking for the target electrogram after loss of PN capture.

## Conclusion

Use of intrapericardial injection of saline, in combination with a steerable epicardial sheath, is a safe and feasible method to prevent PN damage in case of need for epicardial RF ablation at sites with PN capture.
